# Blood Metabolome Mediates the Effect of the Plasma Lipidome on the Risk of Atrial Fibrillation: A Mendelian Randomization Study

**DOI:** 10.1002/clc.70112

**Published:** 2025-03-07

**Authors:** Yanglulu Su, Yi Ning, Zhiyuan Jiang, Guoqiang Zhong

**Affiliations:** ^1^ Department of Cardiology The First Affiliated Hospital of Guangxi Medical University Nanning 530021 China

**Keywords:** atrial fibrillation, blood metabolome, mediation analysis, Plasma lipidome, two‐sample mendelian randomization

## Abstract

**Background and Objective:**

Atrial fibrillation (AF), a common arrhythmic disorder, is increasing in prevalence annually and has become an important public health problem that jeopardizes human health. Metabolites are small molecules produced in the process of metabolic reactions, and they can affect the risk of disease and possibly become targets for disease management.

**Methods:**

We used two‐sample and bidirectional MR to explore potential causal associations between lipid groups and AF. Two‐step MR analysis was used to explore whether plasma metabolites mediated a causal effect from lipidomes to AF.

**Result:**

We assessed the effect of 179 lipids on AF using IVW models and observed that 8 lipids were associated significantly with AF (*p* < 0.05). Likewise, we assessed the effect of 1091 metabolites and 309 metabolite ratios on AF and observed that 22 metabolites were significantly associated with AF (*p* < 0.05). We analyzed the blood metabolites above as mediators in the pathway from the lipidomes above to AF. We found that levels. Of lipid sterol ester (27:1/18:3) were associated with lower homoarginine levels, and lower metabolite homoarginine levels were associated with an increased risk of AF.

**Conclusion:**

Our study identified a causal relationship between plasma liposomes and AF, and additionally found that the plasma metabolite homoarginine levels can act as a mediator of the lipid sterol ester in its effect on AF.

## Graphical abstract

A flowchart for conducting Mendelian randomization analysis.

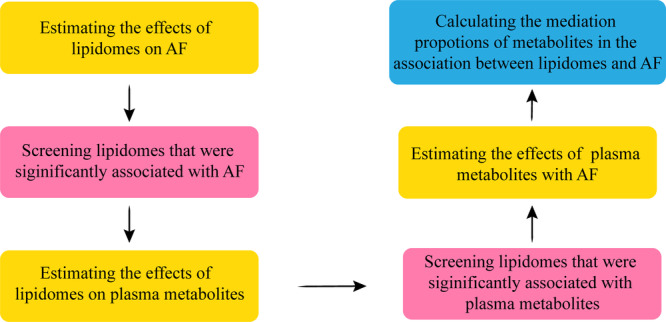

## 1 Introduction

Atrial fibrillation (AF), a common arrhythmic disorder, has become an important research topic in cardiovascular medicine on a global scale. Statistically, the prevalence of AF is increasing every year, making it an important public health problem that jeopardizes human health. According to the Framingham Heart Study (FHS), the prevalence of AF has tripled over the past 50 years [1, 2]. In addition to directly affecting heart function, AF can cause obstruction to the flow of blood through the heart, increasing the risk of thrombus clots in the atria, which can lead to serious complications such as stroke and other serious threats to the patient's life.

Some patients with cardiovascular diseases often experience lipid metabolism disorders, which may lead to changes in atrial structure and function, thereby increasing the incidence of AF. Lipids include many types, including some conventional lipids, such as high‐density lipoprotein cholesterol (HDL‐C), low‐density lipoprotein cholesterol (LDL‐C), triglycerides (TG), and total cholesterol (TC). These lipids have been recognized as risk factors associated with cardiovascular disease. It has been shown that higher levels of HDL‐C and apolipoprotein A‐1 are associated with lower incident primary AF [3]. With the emergence of lipidomics technologies, researchers have gained a deeper understanding of the variability of circulating lipids and lipid classes. Some novel lipids, such as glycerophospholipids, sphingolipids, and sterol lipids, may enhance the assessment of cardiovascular diseases compared to traditional lipids [4, 5]. However, the relevance of these novel lipids to AF is still not known.

Metabolites are small molecular substances produced during metabolic reactions, and their formation and concentration can be easily influenced by factors such as genetics, diet, lifestyle, and disease [6]. In contrast, they can influence disease risk and may become targets for disease treatment [7]. With the continuous development of metabolomics, an increasing number of metabolites have been discovered and studied, providing new insights for a deeper understanding of the mechanisms underlying cardiovascular diseases [8, 9]. Therefore, we have reason to believe that exploring new therapeutic targets for AF at the metabolite level holds significant potential, which may contribute to the development of more effective treatment strategies.

Mendelian randomization (MR) is a potential method of causal inference. MR utilizes strongly correlated single nucleotide polymorphisms (SNP) as an instrumental variable to further investigate the effect of exposure with respect to the outcome [10]. MR reduces the potential for confusion bias and allows for the avoidance of the problem of reverse causality that commonly occurs in observational studies [10].

In this study, we used univariate Mendelian randomization (UVMR) to assess the causal relationship between lipids, metabolites, and AF. A two‐step MR analysis was then used to assess whether these blood metabolites, which are significantly causally associated with AF, could mediate the effect of lipids on the risk of AF. The result is expected to provide more scientific evidence for the prevention, diagnosis, and treatment of AF.

## 2 Methods

### 2.1 Study Design

In this study, we used two‐sample and bidirectional MR to investigate the potential causal association between lipidomes and AF. The GWAS datasets used in this study were all from the European population. We integrated 495 genetic associations related to the lipidomes from the GWAS study by Ottensmann et al. which recruited 7174 Finnish individuals and analyzed 179 lipids [11]. Metabolite statistics were obtained from the GWAS study by Chen et al. in which plasma metabolites from 8299 unrelated European individuals were analyzed [12]. All GWAS data we used are publicly available from the MRC IEU Open GWAS data infrastructure [13] (https://gwas.mrcieu.ac.uk). Therefore, our study did not require ethical approval. According to Emdin et al. three core assumptions need to be followed in MR studies to ensure the validity of potential causality [10]. We used SNP as instrumental variables. MR analyses were performed using R version 4.3.2 and the “Two‐Sample MR” package (version 0.5.6).

### 2.2 Selection of Instrumental Variables

We enrolled genome‐wide significant SNPs (*P* < 5 × 10^−8^) as instrumental variables. To mitigate the effects of linkage disequilibrium (LD), the threshold for SNPs independence was set to *r* ^2^ < 0.001 with an aggregation distance of 10,000 kb. We calculated the F‐statistic using R2 (the proportion of phenotypic variance explained by each SNP) and excluded the instrumental variables with F‐statistic less than 10. [14] Finally, a query was performed using a pheno‐scanner to remove the presence of confounding SNPs.

### 2.3 Mendelian Randomization Analysis

We performed bidirectional and two‐sample MR analyses to assess the causal relationship between the lipid group and AF with total effects obtained. We used MR methods including inverse variance weighted (IVW), MR Egger, weighted median, simple mode, and weighted mode [15, 16, 17]. Various methods were employed to test the validity of the hypotheses. MR Egger was utilized to evaluate the directed pleiotropy of instrumental variables, with the intercept providing an estimate of mean pleiotropy. The IVW method was used to estimate causal effect values when there was no horizontal pleiotropy. The remaining methods are complementary to MR, which provides reliable causal effects when the β values are in the same direction [18].

**Figure 1:**
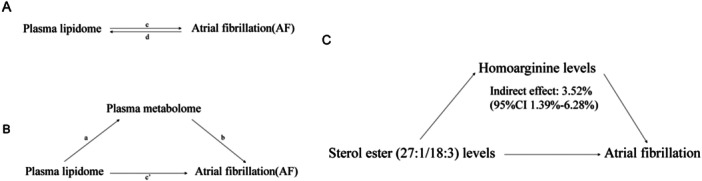
(A, B) Diagrams illustrating associations examined in this study. The total effect between plasma lipidome and atrial fibrillation (AF). c is the total effect using genetically predicted plasma lipidome as exposure and AF as outcome. d is the total effect using genetically predicted AF as exposure and plasma lipidome as outcome. (B) The total effect was decomposed into: (I) indirect effect using a two‐step approach (where a is the total effect of plasma lipidome on plasma metabolome, and b is the effect of plasma metabolome on AF) and the product method (a × b) and (II) direct effect (c′ = c – a × b). Proportion mediated was the indirect effect divided by the total effect. (C) Schematic diagram of the homoarginine levels mediation effect.

We further used two‐step MR analysis to explore whether plasma metabolites mediated a causal effect from lipidomes to AF. The overall effect was categorized into indirect (mediated) and direct (unmediated) effects [19](as shown in Figure 1A,B).

### 2.4 Sensitivity Analysis

Heterogeneity among genetic variants was assessed using Cochran's Q test and funnel plot. Horizontal pleiotropy was then assessed using the MR Egger intercept method [19, 20]. The MR‐PRESSO method was primarily utilized to identify potential outliers and recompute the estimates by excluding them to assess the presence of directional pleiotropy. In cases where heterogeneity is present, the outliers are eliminated, and the causal effect analysis is conducted once more. If heterogeneity still exists after removal, a random effects model is used to assess the robustness of the results [21]. A leave‐one‐out sensitivity analysis is also performed to verify that the causal effects are stable and reliable.

## 3 Results

### 3.1 Relationship Between Lipidomes and AF

First, SNPs with an *F*‐value less than 10 were excluded. Next, the cascade imbalance effect was removed (*r* ^2^ < 0.001 within 10,000 kb). A query using pheno‐scanner was performed to remove SNPs with confounding, and finally, 32 SNPs in the lipid group were used as instrumental variables. We assessed the effect of 179 lipids on AF using the IVW model and observed that 8 lipids were significantly associated with AF (*p* < 0.05). In the MR analysis (Table 1A), we found that lipids phosphatidylcholine (18:2_0:0) levels, phosphatidylcholine (18:0_18:2) levels, phosphatidylinositol (18:0_20:3) levels. phosphatidylcholine (18:1_18:2) levels were negatively correlated with AF (OR 0.8971, 0.9516, 0.9612, 0.9647, 0.9712, *p* < 0.05). On the other hand, phosphatidylcholine (18:1_20:2) levels, phosphatidylcholine (18:2_20:4) levels, sterol ester (27:1/18:3) levels, sterol ester (27:1/16:1) levels were positively correlated with AF (OR 1.0647, 1.0661, 1.1331, respectively, *p* < 0.05). The effects of these lipids on AF were not heterogeneous (P‐heterogeneity > 0.05) or pleiotropic (P‐intercept > 0.05) across instruments. In performing the inverse MR analysis, we found that there was no inverse causality between AF and any of the above‐mentioned lipids, and the results are shown in Table 1B.

**Table 1A:** MR estimates of the effect of lipidomes on atrial fibrillation (AF).

Exposure	Outcome	SNPs	Method	OR (95% CI)	*P*	Q statistic	*P*‐heterogeneity	Egger intercept	*P*‐intercept
Sterol ester (27:1/16:1) levels	AF	3	IVW	1.1331 (1.0210, 1.2575)	0.0187	2.5380	0.2811		
			MR‐Egger	1.2450 (0.3910, 3.9643)	0.7740	2.4741	0.1157	−0.0160	0.8986
			Weighted median	1.1147 (1.0012, 1.2410)	0.0474				
			Simple mode	1.0985 (0.9710, 1.2426)	0.2741				
			Weighted mode	1.1129 (0.9973, 1.2420)	0.1962				
Sterol ester (27:1/18:3) levels	AF	5	IVW	1.0661 (1.0101, 1.1253)	0.0202	4.0679	0.3969		
			MR‐Egger	1.1379 (0.9594, 1.3496)	0.2344	3.3615	0.3392	−0.0098	0.4852
			Weighted median	1.0710 (1.0058, 1.1405)	0.0324				
			Simple mode	1.0749 (0.9892, 1.1680)	0.1636				
			Weighted mode	1.0740 (1.0013, 1.1520)	0.1167				
Phosphatidylcholine (18:2_0:0) levels	AF	3	IVW	0.8971 (0.8286, 0.9712)	0.0073	1.6348	0.4416		
			MR‐Egger	0.5773 (0.2798, 1.1914)	0.3771	0.1957	0.6582	0.0564	0.4424
			Weighted median	0.9080 (0.8292, 0.9943)	0.0372				
			Simple mode	0.9112 (0.8226, 1.0094)	0.2169				
			Weighted mode	0.9087 (0.8266, 0.9990)	0.1860				
Phosphatidylcholine (18:0_18:2) levels	AF	5	IVW	0.9516 (0.9131, 0.9918)	0.0188	1.8730	0.7591		
			MR‐Egger	0.9358 (0.8255, 1.0609)	0.3761	1.7959	0.6158	0.0031	0.7992
			Weighted median	0.9439 (0.8976, 0.9927)	0.0248				
			Simple mode	0.9419 (0.8736, 1.0156)	0.1944				
			Weighted mode	0.9434 (0.8967, 0.9925)	0.0875				
Phosphatidylcholine (18:1_18:2) levels	AF	5	IVW	0.9647 (0.9348, 0.9956)	0.0254	4.2333	0.3754		
			MR‐Egger	0.9670 (0.9022, 1.0364)	0.4122	4.2249	0.2382	−0.0006	0.9435
			Weighted median	0.9653 (0.9324, 0.9993)	0.0454				
			Simple mode	0.9391 (0.8779, 1.0045)	0.1415				
			Weighted mode	0.9662 (0.9335, 1.0001)	0.1226				
Phosphatidylcholine (18:1_20:2) levels	AF	3	IVW	0.9712 (0.9433, 0.9999)	0.0489	0.2383	0.8877		
			MR‐Egger	0.9801 (0.9173, 1.0471)	0.6575	0.1479	0.7006	−0.0032	0.8141
			Weighted median	0.9724 (0.9444, 1.0012)	0.0601				
			Simple mode	0.9755 (0.9292, 1.0242)	0.4237				
			Weighted mode	0.9725 (0.9419, 1.0041)	0.2291				
Phosphatidylcholine (18:2_20:4) levels	AF	4	IVW	1.0647 (1.0005, 1.1331)	0.0483	3.5610	0.3129		
			MR‐Egger	1.1559 (0.8587, 1.5559)	0.4403	3.0815	0.2142	−0.0115	0.6330
			Weighted median	1.0560 (0.9859, 1.1310)	0.1199				
			Simple mode	1.0339 (0.9437, 1.1328)	0.5254				
			Weighted mode	1.0585 (0.9840, 1.1386)	0.2244				
Phosphatidylinositol (18:0_20:3) levels	AF	4	IVW	0.9612 (0.9295, 0.9940)	0.0209	1.6576	0.6464		
			MR‐Egger	0.9583 (0.8846, 1.0381)	0.4062	1.6509	0.4380	0.0007	0.9423
			Weighted median	0.9626 (0.9284, 0.9980)	0.0388				
			Simple mode	0.9893 (0.9314, 1.0508)	0.7504				
			Weighted mode	0.9623 (0.9237, 1.0024)	0.1625				

**Table 1B:** Reverse MR analyses of the relationship between lipidomes and AF.

Exposure	Outcome	SNPs	Method	OR (95% CI)	*P*
AF	Sterol ester (27:1/16:1) levels	107	IVW	0.9943 (0.9464, 1.0447)	0.8221
			MR‐Egger	0.9439 (0.8593, 1.0370)	0.2316
			Weighted median	0.9585 (0.8825, 1.0410)	0.3141
			Simple mode	1.0066 (0.8208, 1.2344)	0.9500
			Weighted mode	0.9164 (0.8317, 1.0099)	0.0810
AF	Sterol ester (27:1/18:3) levels	107	IVW	1.0003 (0.9527, 1.0504)	0.9890
			MR‐Egger	0.9754 (0.8884, 1.0710)	0.6027
			Weighted median	0.9358 (0.8646, 1.0130)	0.1008
			Simple mode	0.9205 (0.7374, 1.1491)	0.4659
			Weighted mode	0.9076 (0.8322, 0.9898)	0.0306
AF	Phosphatidylcholine (18:2_0:0) levels	107	IVW	0.9986 (0.9439, 1.0565)	0.9619
			MR‐Egger	1.0895 (0.9797, 1.2116)	0.1167
			Weighted median	0.9751 (0.8964, 1.0608)	0.5576
			Simple mode	1.0289 (0.8708, 1.2157)	0.7386
			Weighted mode	1.0050 (0.9223, 1.0950)	0.9103
AF	Phosphatidylcholine (18:0_18:2) levels	107	IVW	1.0284 (0.9724, 1.0876)	0.3268
			MR‐Egger	1.1259 (1.0132, 1.2510)	0.0297
			Weighted median	1.0530 (0.9635, 1.1509)	0.2543
			Simple mode	1.0782 (0.9102, 1.2773)	0.3855
			Weighted mode	1.0851 (0.9910, 1.1881)	0.0803
AF	Phosphatidylcholine (18:1_18:2) levels	107	IVW	1.0205 (0.9609, 1.0837)	0.5093
			MR‐Egger	1.1127 (0.9931, 1.2466)	0.0685
			Weighted median	0.9890 (0.9069, 1.0785)	0.8028
			Simple mode	0.9773 (0.7800, 1.2244)	0.8419
			Weighted mode	1.0345 (0.9377, 1.1413)	0.5002
AF	Phosphatidylcholine (18:1_20:2) levels	107	IVW	1.0090 (0.9530, 1.0682)	0.7583
			MR‐Egger	1.0431 (0.9353, 1.1635)	0.4498
			Weighted median	0.9875 (0.8955, 1.0891)	0.8016
			Simple mode	1.1859 (0.9578, 1.4682)	0.1207
			Weighted mode	1.0384 (0.9369, 1.1510)	0.4742
AF	Phosphatidylcholine (18:2_20:4) levels	107	IVW	0.9884 (0.9399, 1.0393)	0.6486
			MR‐Egger	1.0808 (0.9835, 1.1876)	0.1095
			Weighted median	0.9886 (0.9034, 1.0818)	0.8030
			Simple mode	0.8835 (0.7377, 1.0581)	0.1811
			Weighted mode	0.9900 (0.9070, 1.0807)	0.8232
AF	Phosphatidylinositol (18:0_20:3) levels	107	IVW	1.0145 (0.9652, 1.0663)	0.5721
			MR‐Egger	0.9674 (0.8796, 1.0638)	0.4952
			Weighted median	0.9608 (0.8866, 1.0413)	0.3300
			Simple mode	1.0544 (0.8766, 1.2684)	0.5751
			Weighted mode	0.9670 (0.8804, 1.0622)	0.4854

### 3.2 Relationship Between Lipids and Plasma Metabolites

After excluding SNPs with *F*‐values less than 10 and removing the cascade disequilibrium effect (*r* ^2^ < 0.001 within 10,000 kb), a query was performed using pheno‐scanner to remove SNPs with confounding, and 11 SNPs were used as instrumental variables in lipids. In MR analysis (Table 2B), lipid sterol ester (27:1/18:3) levels were negatively correlated with Homoarginine levels, (OR 0.7919, *p* < 0.05). The effect of this lipid on metabolites was not heterogeneous (P‐heterogeneity > 0.05) or pleiotropic (P‐intercept > 0.05) between instruments.

**Table 2A:** MR estimates of the effect of blood metabolites on AF.

Exposure	Outcome	SNPs	Method	OR (95% CI)	*P*	Q statistic	*P*‐heterogeneity	Egger intercept	*P*‐intercept
Homoarginine levels	AF	5	IVW	0.9558 (0.9184, 0.9947)	0.0263	4.6930	0.3203		
			MR‐Egger	0.9403 (0.8638, 1.0235)	0.2501	4.4037	0.2210	0.0044	0.6872
			Weighted median	0.9570 (0.9161, 0.9996)	0.0479				
			Simple mode	0.9400 (0.8844, 0.9992)	0.1178				
			Weighted mode	0.9541 (0.9069, 1.0038)	0.1439				
5‐methyluridine (ribothymidine) levels	AF	3	IVW	0.9711 (0.9457, 0.9971)	0.0294	0.4336	0.8051		
			MR‐Egger	0.9711 (0.9319, 1.0120)	0.3964	0.4336	0.5102	0.0001	0.9980
			Weighted median	0.9707 (0.9449, 0.9972)	0.0305				
			Simple mode	0.9654 (0.9281, 1.0043)	0.2228				
			Weighted mode	0.9710 (0.9436, 0.9993)	0.1821				
Hexadecanedioate (C16‐DC) levels	AF	5	IVW	0.9607 (0.9326, 0.9896)	0.0081	1.0438	0.9031		
			MR‐Egger	0.9493 (0.9038, 0.9970)	0.1289	0.6833	0.8771	0.0038	0.5906
			Weighted median	0.9586 (0.9285, 0.9896)	0.0093				
			Simple mode	0.9613 (0.9096, 1.0159)	0.2338				
			Weighted mode	0.9576 (0.9271, 0.9892)	0.0588				
Indoleacetylglutamine levels	AF	4	IVW	1.0644 (1.0147, 1.1166)	0.0105	3.2409	0.3560		
			MR‐Egger	1.3256 (0.9778, 1.7971)	0.2111	1.1969	0.5497	‐0.0441	0.2891
			Weighted median	1.0559 (0.9991, 1.1159)	0.0537				
			Simple mode	1.0470 (0.9739, 1.1257)	0.3019				
			Weighted mode	1.0570 (0.9957, 1.1221)	0.1665				
5alpha‐pregnan‐diol disulfate levels	AF	3	IVW	0.9511 (0.9104, 0.9937)	0.0247	0.2879	0.8660		
			MR‐Egger	0.9076 (0.7606, 1.0830)	0.4769	0.0004	0.9833	0.0134	0.6867
			Weighted median	0.9534 (0.9090, 1.0001)	0.0506				
			Simple mode	0.9678 (0.9083, 1.0312)	0.4185				
			Weighted mode	0.9435 (0.8970, 0.9925)	0.1530				
Pregnenolone sulfate levels	AF	7	IVW	0.9163 (0.8540, 0.9831)	0.0149	12.2718	0.0562		
			MR‐Egger	0.9614 (0.7975, 1.1590)	0.6968	11.5729	0.0411	‐0.0069	0.6063
			Weighted median	0.8927 (0.8398, 0.9488)	0.0003				
			Simple mode	0.8956 (0.8276, 0.9692)	0.0339				
			Weighted mode	0.8930 (0.8361, 0.9537)	0.0150				
N‐acetylcarnosine levels	AF	5	IVW	0.9442 (0.9069, 0.9829)	0.0051	2.9243	0.5706		
			MR‐Egger	0.8689 (0.7558, 0.9988)	0.1426	1.4349	0.6974	0.0144	0.3095
			Weighted median	0.9394 (0.8959, 0.9849)	0.0096				
			Simple mode	0.9335 (0.8780, 0.9924)	0.0922				
			Weighted mode	0.9339 (0.8851, 0.9855)	0.0674				
2‐aminooctanoate levels	AF	3	IVW	0.9717 (0.9449, 0.9992)	0.0436	0.4748	0.7887		
			MR‐Egger	0.9699 (0.9148, 1.0283)	0.4919	0.4699	0.4930	0.0009	0.9553
			Weighted median	0.9707 (0.9435, 0.9986)	0.0400				
			Simple mode	0.9642 (0.9099, 1.0217)	0.3426				
			Weighted mode	0.9705 (0.9430, 0.9989)	0.1786				
1‐oleoyl‐GPG (18:1) levels	AF	3	IVW	0.9514 (0.9187, 0.9852)	0.0052	0.3795	0.8272		
			MR‐Egger	0.9446 (0.8872, 1.0058)	0.3259	0.3071	0.5795	0.0022	0.8327
			Weighted median	0.9496 (0.9157, 0.9848)	0.0053				
			Simple mode	0.9451 (0.8969, 0.9960)	0.1693				
			Weighted mode	0.9493 (0.9133, 0.9867)	0.1188				
Octadecenedioylcarnitine (C18:1‐DC) levels	AF	4	IVW	0.9619 (0.9354, 0.9892)	0.0064	0.7560	0.8600		
			MR‐Egger	0.9511 (0.9082, 0.9961)	0.1672	0.3947	0.8209	0.0041	0.6088
			Weighted median	0.9591 (0.9311, 0.9879)	0.0058				
			Simple mode	0.9596 (0.9169, 1.0044)	0.1745				
			Weighted mode	0.9590 (0.9325, 0.9862)	0.0606				
Taurodeoxycholic acid 3‐sulfate levels	AF	5	IVW	1.0344 (1.0028, 1.0670)	0.0325	3.0615	0.4767		
			MR‐Egger	1.0025 (0.9189, 1.0937)	0.9585	2.4923	0.5476	0.0087	0.5054
			Weighted median	1.0298 (0.9964, 1.0643)	0.0805				
			Simple mode	1.0285 (0.9754, 1.0844)	0.3578				
			Weighted mode	1.0285 (0.9930, 1.0652)	0.1919				
Glycodeoxycholate 3‐sulfate levels	AF	5	IVW	0.9642 (0.9389, 0.9901)	0.0070	2.3997	0.6627		
			MR‐Egger	0.9389 (0.8931, 0.9871)	0.0901	0.8948	0.8267	0.0089	0.3074
			Weighted median	0.9590 (0.9308, 0.9882)	0.0061				
			Simple mode	0.9663 (0.9291, 1.0050)	0.1625				
			Weighted mode	0.9593 (0.9306, 0.9889)	0.0552				
1‐stearoyl‐2‐linoleoyl‐gpc (18:0/18:2) levels	AF	3	IVW	0.9415 (0.8933, 0.9924)	0.0248	1.2258	0.5418		
			MR‐Egger	0.9688 (0.8507, 1.1034)	0.7163	1.0037	0.3164	‐0.0049	0.7201
			Weighted median	0.9477 (0.8967, 1.0016)	0.0569				
			Simple mode	0.9612 (0.8811, 1.0486)	0.4666				
			Weighted mode	0.9515 (0.8969, 1.0095)	0.2412				
1‐stearoyl‐2‐arachidonoyl‐GPE (18:0/20:4) levels	AF	7	IVW	1.0298 (1.0019, 1.0586)	0.0364	7.9953	0.2384		
			MR‐Egger	1.0320 (0.9488, 1.1225)	0.4957	7.9910	0.1567	‐0.0006	0.9606
			Weighted median	1.0242 (0.9941, 1.0551)	0.1165				
			Simple mode	1.0149 (0.9667, 1.0656)	0.5730				
			Weighted mode	1.0324 (1.0003, 1.0655)	0.0956				
N‐acetyl‐2‐aminooctanoate levels	AF	4	IVW	1.0288 (1.0012, 1.0571)	0.0407	0.1931	0.9787		
			MR‐Egger	1.0376 (0.9677, 1.1126)	0.4084	0.1251	0.9394	‐0.0043	0.8186
			Weighted median	1.0271 (1.0015, 1.0513)	0.0416				
			Simple mode	1.0287 (0.9670, 1.0943)	0.4364				
			Weighted mode	1.0295 (1.0007, 1.0592)	0.1385				
N‐acetyl‐isoputreanine levels	AF	7	IVW	0.9423 (0.8908, 0.9968)	0.0381	22.7049	0.0009		
			MR‐Egger	0.8522 (0.7363, 0.9863)	0.0847	16.0381	0.0067	0.0233	0.2090
			Weighted median	0.9242 (0.8903, 0.9594)	0.0000				
			Simple mode	0.9254 (0.8811, 0.9719)	0.0212				
			Weighted mode	0.9200 (0.8858, 0.9556)	0.0050				
Lithocholate sulfate (1) levels	AF	3	IVW	0.9363 (0.8831, 0.9927)	0.0275	2.6349	0.2678		
			MR‐Egger	0.9454 (0.6684, 1.3373)	0.8044	2.6266	0.1051	‐0.0018	0.9643
			Weighted median	0.9583(0.8992, 1.0212)	0.1892				
			Simple mode	0.9654 (0.8793, 1.0600)	0.5376				
			Weighted mode	0.9697 (0.8829, 1.0650)	0.5856				
Deoxycholic acid 12‐sulfate levels	AF	4	IVW	0.9591 (0.9224, 0.9973)	0.0361	3.9954	0.2620		
			MR‐Egger	0.9384 (0.8132, 1.0828)	0.4758	3.8051	0.1492	0.0066	0.7818
			Weighted median	0.9473 (0.9118, 0.9842)	0.0055				
			Simple mode	0.9500 (0.8986, 1.0043)	0.1681				
			Weighted mode	0.9462 (0.9067, 0.9874)	0.0843				
Metabolonic lactone sulfate levels	AF	7	IVW	0.9670 (0.9409, 0.9938)	0.0160	4.2074	0.6486		
			MR‐Egger	0.9701 (0.9179, 1.0252)	0.3311	4.1898	0.5224	‐0.0009	0.8996
			Weighted median	0.9544 (0.9217, 0.9882)	0.0086				
			Simple mode	0.9522 (0.8978, 1.0098)	0.1532				
			Weighted mode	0.9514 (0.9171, 0.9869)	0.0372				
1‐palmitoyl‐2‐linoleoyl‐gpc (16:0/18:2) levels	AF	4	IVW	0.9502 (0.9105, 0.9918)	0.0193	2.1404	0.5438		
			MR‐Egger	0.9588 (0.8757, 1.0498)	0.4593	2.0891	0.3518	‐0.0017	0.8452
			Weighted median	0.9519 (0.9098, 0.9959)	0.0327				
			Simple mode	0.9378 (0.8645, 1.0174)	0.2200				
			Weighted mode	0.9527 (0.9065, 1.0012)	0.1520				
N‐acetylputrescine levels	AF	4	IVW	1.0481 (1.0224, 1.0743)	0.0002	1.2339	0.7449		
			MR‐Egger	1.0213 (0.9639, 1.0821)	0.5491	0.2954	0.8627	0.0133	0.4349
			Weighted median	1.0461 (1.0196, 1.0733)	0.0006				
			Simple mode	1.0891 (1.0320, 1.1494)	0.0530				
			Weighted mode	1.0452 (1.0175, 1.0735)	0.0481				
X‐24546 levels	AF	4	IVW	0.9458 (0.9095, 0.9835)	0.0052	0.8990	0.8257		
			MR‐Egger	0.9405 (0.8617, 1.0265)	0.3030	0.8794	0.6442	0.0015	0.9014
			Weighted median	0.9422 (0.9031, 0.9831)	0.0060				
			Simple mode	0.9405 (0.8830, 1.0018)	0.1532				
			Weighted mode	0.9405 (0.8991, 0.9839)	0.0758				

**Table 2B:** MR estimates of the effect of lipidomes on blood metabolites.

Exposure	Outcome	SNPs	Method	OR (95% CI)	*P*	Q statistic	*P*‐heterogeneity	Egger intercept	*P*‐intercept
Sterol ester (27:1/18:3) levels	Homoarginine levels	5	IVW	0.7677 (0.6724, 0.8765)	0.0001	2.2887	0.6828		
			MR‐Egger	0.7404 (0.4994, 1.0976)	0.2314	2.2520	0.5218	0.0055	0.8603
			Weighted median	0.7709 (0.6527, 0.9107)	0.0022				
			Simple mode	0.7560 (0.6000, 0.9524)	0.0765				
			Weighted mode	0.7698 (0.6363, 0.9313)	0.0545				

### 3.3 Relationship Between Plasma Metabolites and AF

Excluding SNPs with *F*‐values less than 10 as well as removing the cascade imbalance effect (*r* ^2^ < 0.001 within 10,000 kb), a query was performed using pheno‐scanner to remove SNPs with confounding, and 104 SNPs in metabolites were used as instrumental variables. We assessed the effect of 1091 metabolites and 309 metabolite ratios on AF using the IVW model and observed that 22 metabolites were significantly associated with AF (*p* < 0.05). In the MR analysis (Table 2A), we observed that homoarginine levels, 5‐methyluridine (ribothymidine) levels, hexadecanedioate (C16‐DC) levels, 5alpha‐pregnan‐diol disulfate levels, pregnenolone sulfate levels, N‐acetylcarnosine levels, 2‐aminooctanoate levels, 1‐oleoyl‐GPG (18:1) levels. octadecenedioylcarnitine (C18:1‐DC) levels, glycodeoxycholate 3‐sulfate levels, 1‐stearoyl‐2‐linoleoyl‐gpc(18:0/18:2) levels, N‐acetyl‐ isoputreanine levels, lithocholate sulfate (1) levelss, deoxycholic acid 12‐sulfate levels, metabolonic lactone sulfate levels, 1‐palmitoyl‐2‐ linoleoyl‐gpc (16:0/18:2) levels, and X‐24546 levels were negatively correlated with AF (OR 0.9558, 0.9711, 0.9607, 0.9511, 0.9163, 0.9442, 0.9717, 0.9514, 0.9619, 0.9642, 0.9415, 0.9415, 0.9415, 0.9415, 0.9415, 0.9415, 0.9415, 0.9415, 0.9415, 0.9415, 0.9415, 0.9642 0.9415, 0.9423, 0.9363, 0.9591, 0.9670, 0.9502, 0.9458, *p* < 0.05), while indoleacetylglutamine levels, taurodeoxycholic acid 3‐sulfate levels, N‐ acetyl‐2‐aminooctanoate levels, N‐acetylputrescine levels were positively correlated with AF (OR 1.0644, 1.0344, 1.0288, and 1.0481, *p* < 0.05). The effects of these metabolites and metabolite ratios on AF were not heterogeneous across instruments (P‐heterogeneity > 0.05) or pleiotropic (P‐intercept > 0.05).

### 3.4 Mendelian Randomization of Mediators

**Figure 2:**
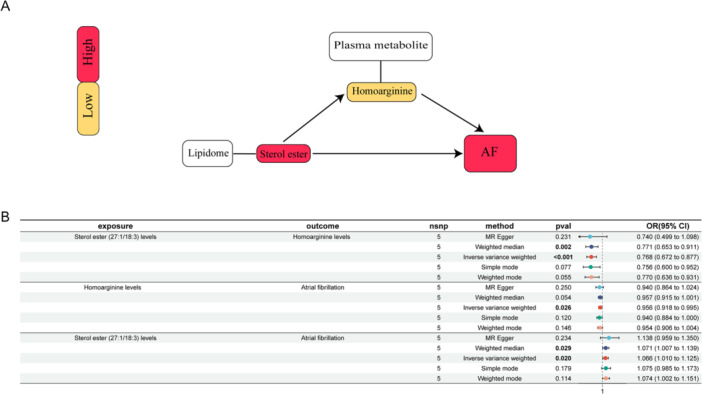
(A) A diagram showing the relationship between sterol esters, homoarginine, and AF. AF, atrial fibrillation. (B) Forest plot to visualize the causal effects of homoarginine levels with sterol ester (27:1/18:3) levels and AF.

**Figure 3:**
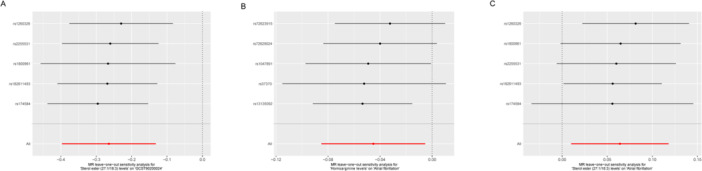
Sensitivity analyses using the leave‐one‐out approach on the association of exposures on outcome. (A) sterol ester (27:1/18:3) levels‐homoarginine levels; (B) homoarginine levels‐AF; (C) sterol ester (27:1/18:3) levels‐AF. Each black dot represents an IVW method for estimating causal the effect of the exposures on the AF does not exclude a case where a particular SNP caused a significant change in the overall results. AF, atrial fibrillation; SNP, single‐nucleotide polymorphism.

We analyzed the above blood metabolites as mediators of the pathway from the above liposome to AF, and we found that lipid sterol ester (27:1/18:3) levels were associated with lower homoarginine levels, and lower metabolite homoarginine levels were associated with an increased risk of AF (Figure 2B). Homoarginine levels accounted for 3.52% (95% CI 1.39–6.28%) of the association between sterol ester (27:1/18:3) levels and AF risk, as shown in Figure 1C. Sensitivity analyses using the leave‐one‐out method confirmed the causal association between homoarginine levels and sterol ester (27:1/18:3) levels and the risk of AF (Figure 3A,B), and the homoarginine levels and sterol ester (27:1/18:3) levels (Figure 3C).

## 4 Discussion

This study is the first to investigate the relationship among lipids, metabolites, and AF using MR combined with mediation analysis. The findings reveal that eight lipids have a potential causal relationship with AF, while 22 metabolites are associated with the condition. Subsequent mediation MR analysis indicated that the level of the metabolite homoarginine may mediate the relationship between the lipid sterol ester (27:1/18:3) and AF. However, no significant mediating effects were observed for the other lipids and metabolites in their impact on AF.

Phosphatidylcholine is a phospholipid compound found in both animal and plant cell membranes, plays a crucial role in cellular signaling pathways, and is abundant in daily dietary intake. When foods rich in phosphatidylcholine enter the intestine, trimethylamine‐N‐oxide (TMAO) is produced by the gut microbiota. Elevated levels of TMAO have been positively correlated with atherosclerosis, ischemic stroke, as TMAO increases macrophage cholesterol accumulation and foam cell formation [22]. Therefore, in clinical practice, when advising patients with AF on dietary choices, it may be beneficial to consider reducing the intake of TMAO to lower the risk of AF‐related complications.

The study by Li et al. found that an increase in carbon chain length and a decrease in saturation of phosphatidylcholine are associated with a higher risk of AF, which is consistent with our findings. Furthermore, our study provides evidence for a potential unidirectional causal relationship between phosphatidylcholine and AF [23]. In studies of inflammatory bowel disease (IBD), it has been found that phosphatidylcholine levels are reduced in the intestinal mucosa of patients with IBD, that insufficient dietary choline exacerbates the condition, and that supplementation with choline reduces the prevalence of colitis in mice [24]. In mice, the process is mainly *Escherichia coli* through choline, upregulation of transforming growth factor‐β (TGF‐β), and induction of cytokine immunotolerance to T cells, leading to the exertion of anti‐inflammatory properties. This suggests that moderate supplementation of phosphatidylcholine in dietary interventions for patients with AF may have potential benefits, although further research is necessary.

Despite phosphatidylinositol accounting for a minor proportion of cellular membrane phospholipids (less than 5%), it plays a crucial role in maintaining cellular functions. Soybeans are rich in phosphatidylinositol, and their consumption can elevate HDL‐C levels and apolipoprotein A‐1 levels, while reducing triglycerides, thereby offering cardiovascular protection. Phosphatidylinositol also participates in the phosphatidylinositol 3‐kinase (PI3K)/Akt signaling pathway, enhancing this signaling cascade to improve arrhythmias [25].

Sterol esters, as important components of cell membranes and hormone synthesis, are closely associated with the occurrence of diseases such as atherosclerosis and diabetes when they accumulate abnormally [26]. Our study found that elevated levels of sterol esters may be associated with an increased risk of AF, suggesting that monitoring sterol ester levels in clinical practice could be beneficial for assessing the risk of AF.

The application of metabolomics provides us with new opportunities for investigating potential biomarkers for AF. In this study, various metabolites were found to have causal relationships with AF, with some metabolites confirmed in previous studies to be associated with multiple diseases. Hexadecanedioate (C16‐DC) levels serve as biomarkers for transporter proteins [27]. Pregnenolone sulfate levels, an endogenous neurosteroid, are mainly associated with cognitive aging and sleep regulation in humans, offering potential as a new type of neurotherapeutic drug [28]. N‐acetylcarnosine levels, an antioxidant, have been shown to improve or reverse lens opacities in patients with cataracts [29]. Metabolonic lactone sulfate levels are associated with metabolic syndrome and various characteristics of heart metabolic diseases, such as glucose homeostasis and total triglyceride levels [30]. Some metabolites’ functions are yet to be confirmed, and this study introduces a new possibility of exploring potential therapeutic targets for AF by investigating the metabolites associated with it.

Another metabolite with a potential causal link to AF, homoarginine, is an important non‐protein amino acid. Research has shown that elevated levels of homoarginine can inhibit the activity of arginase, leading to increased levels of arginine and nitric oxide, thereby improving vascular status [31]. Lower levels of homoarginine are associated with the occurrence and progression of AF and may exert a protective effect by influencing the activity of T cells [32]. Specifically, our study found that the level of sterol ester (27:1/18:3) influences the progression of AF by affecting homoarginine level (Figure 2A). Homoarginine may become a new target for future AF treatment, and the potential effects of homoarginine supplementation merit validation in clinical trials.

This study has certain limitations. First, the MR method we employed relies on several assumptions, including the validity and independence of the instrumental variables. However, the effectiveness of MR analysis can be affected by the relationship between the instrumental variables and potential confounding factors, which may lead to biased results. In addition, although our use of a univariate model helps control for the risk of overfitting present in multivariate models and appropriate instrumental variables were selected to minimize confounding effects, other unmeasured variables that may influence AF risk still need to be considered. Furthermore, while our sample size is relatively large and encompasses various populations, there may still be issues of selection bias, particularly due to differences in participant selection and sample sources, which may limit the generalizability of our findings to broader populations. Finally, although this study provides some causal inference using the MR approach, further large‐scale randomized controlled trials are needed to validate the exact roles and mechanisms of metabolites and lipids in the occurrence of AF.

## 5 Conclusion

Our research has elucidated the causal relationship between plasma lipids and AF, and we have also discovered that plasma metabolite homoarginine levels can act as a mediator for the impact of lipid sterol ester on AF. The mediating effect accounts for a relatively small proportion, while the mechanisms by which lipids influence AF remain unclear. Further research is needed to confirm the effects of homoarginine levels and other potential mediators on AF.

## Author Contributions

YS and YN conceived and designed the study and performed the statistical analysis, GZ revised the first draft of the paper and was primarily responsible for the final content, YS, LN, and ZJ wrote the first draft of the paper. All authors read and approved the final manuscript.

## Conflicts of Interest

The authors declare no conflicts of interest.

## Data Availability

The GWAS Summary statistics used in this study were publicly accessed from the IEU OpenGWAS project (https://gwas.mrcieu.ac.uk/), the GWAS Catalog (https://www.ebi.ac.uk/gwas/), plasma lipidomes code GCST0277238‐GCST90277416, blood metabolomes code GCST90199621‐GCST90204063.

## References

[1] J. Kornej , C. S. Börschel , E. J. Benjamin , and R. B. Schnabel , “Epidemiology of Atrial Fibrillation in the 21st Century: Novel Methods and New Insights,” Circulation Research 127, no. 1 (June 2020): 4–20, 10.1161/circresaha.120.316340. 32716709 PMC7577553

[2] S. Shi , Y. Tang , Q. Zhao , et al., “Prevalence and Risk of Atrial Fibrillation in China: A National Cross‐Sectional Epidemiological Study,” Lancet Regional Health ‐ Western Pacific 23 (June 2022): 100439, 10.1016/j.lanwpc.2022.100439. 35800039 PMC9252928

[3] B. Tajik , T. P. Tuomainen , R. Jarroch , J. Kauhanen , G. Y. H. Lip , and M. Isanejad , “Lipid Levels, Apolipoproteins, and Risk of Incident Atrial Fibrillation in Men: A Report From the Kuopio Ischaemic Heart Disease Risk Factor Study (KIHD),” Journal of Clinical Lipidology 16, no. 4 (July/August 2022): 447–454, 10.1016/j.jacl.2022.04.003. 35525793

[4] R. Tabassum and S. Ripatti , “Integrating Lipidomics and Genomics: Emerging Tools to Understand Cardiovascular Diseases,” Cellular and Molecular Life Sciences 78, no. 6 (MArch 2021): 2565–2584, 10.1007/s00018-020-03715-4. 33449144 PMC8004487

[5] A. M. Poss , J. A. Maschek , J. E. Cox , et al., “Machine Learning Reveals Serum Sphingolipids as Cholesterol‐Independent Biomarkers of Coronary Artery Disease,” Journal of Clinical Investigation 130, no. 3 (March 2020): 1363–1376, 10.1172/jci131838. 31743112 PMC7269567

[6] M. Pietzner , I. D. Stewart , J. Raffler , et al., “Plasma Metabolites to Profile Pathways in Noncommunicable Disease Multimorbidity,” Nature Medicine 27, no. 3 (March 2021): 471–479, 10.1038/s41591-021-01266-0. PMC8127079 33707775

[7] D. S. Wishart , “Emerging Applications of Metabolomics in Drug Discovery and Precision Medicine,” Nature Reviews Drug Discovery 15, no. 7 (July 2016): 473–484, 10.1038/nrd.2016.32. 26965202

[8] R. W. McGarrah , S. B. Crown , G. F. Zhang , S. H. Shah , and C. B. Newgard , “Cardiovascular Metabolomics,” Circulation Research 122, no. 9 (April 2018): 1238–1258, 10.1161/circresaha.117.311002. 29700070 PMC6029726

[9] J. Lv , C. Pan , Y. Cai , et al., “Plasma Metabolomics Reveals the Shared and Distinct Metabolic Disturbances Associated With Cardiovascular Events in Coronary Artery Disease,” Nature Communications 15, no. 1 (July 2024): 5729, 10.1038/s41467-024-50125-2. PMC11231153 38977723

[10] C. A. Emdin , A. V. Khera , and S. Kathiresan , “Mendelian Randomization,” Journal of the American Medical Association 318, no. 19 (November 2017): 1925–1926, 10.1001/jama.2017.17219. 29164242

[11] L. Ottensmann , R. Tabassum , S. E. Ruotsalainen , et al., “Genome‐Wide Association Analysis of Plasma Lipidome Identifies 495 Genetic Associations,” Nature Communications 14, no. 1 (October 2023): 6934, 10.1038/s41467-023-42532-8. PMC10618167 37907536

[12] Y. Chen , T. Lu , U. Pettersson‐Kymmer , et al., “Genomic Atlas of the Plasma Metabolome Prioritizes Metabolites Implicated in Human Diseases,” Nature Genetics 55, no. 1 (January 2023): 44–53, 10.1038/s41588-022-01270-1. 36635386 PMC7614162

[13] B. Elsworth , M. Lyon , T. Alexander , et al., “The MRC IEU OpenGWAS Data Infrastructure,” bioRxiv (2020), 10.1101/2020.08.10.244293.

[14] S. Burgess and S. G. Thompson , “Avoiding Bias From Weak Instruments in Mendelian Randomization Studies,” International Journal of Epidemiology 40, no. 3 (June 2011): 755–764, 10.1093/ije/dyr036. 21414999

[15] G. Hemani , J. Zheng , B. Elsworth , et al., “The Mr‐Base Platform Supports Systematic Causal Inference Across the Human Phenome,” eLife 7 (May 2018): e34408, 10.7554/eLife.34408. 29846171 PMC5976434

[16] S. Burgess and S. G. Thompson , “Interpreting Findings From Mendelian Randomization Using the MR‐Egger Method,” European Journal of Epidemiology 32, no. 5 (May 2017): 377–389, 10.1007/s10654-017-0255-x. 28527048 PMC5506233

[17] J. Bowden , G. Davey Smith , P. C. Haycock , and S. Burgess , “Consistent Estimation in Mendelian Randomization With Some Invalid Instruments Using a Weighted Median Estimator,” Genetic Epidemiology 40, no. 4 (May 2016): 304–314, 10.1002/gepi.21965. 27061298 PMC4849733

[18] Y. Long , L. Tang , Y. Zhou , S. Zhao , and H. Zhu , “Causal Relationship Between Gut Microbiota and Cancers: A Two‐Sample Mendelian Randomisation Study,” BMC Medicine 21, no. 1 (February 2023): 66, 10.1186/s12916-023-02761-6. 36810112 PMC9945666

[19] A. R. Carter , E. Sanderson , G. Hammerton , et al., “Mendelian Randomisation for Mediation Analysis: Current Methods and Challenges for Implementation,” European Journal of Epidemiology 36, no. 5 (May 2021): 465–478, 10.1007/s10654-021-00757-1. 33961203 PMC8159796

[20] M. Verbanck , C. Y. Chen , B. Neale , and R. Do , “Detection of Widespread Horizontal Pleiotropy in Causal Relationships Inferred From Mendelian Randomization Between Complex Traits and Diseases,” Nature Genetics 50, no. 5 (May 2018): 693–698, 10.1038/s41588-018-0099-7. 29686387 PMC6083837

[21] J. Yuan , X. Xiong , B. Zhang , et al., “Genetically Predicted C‐Reactive Protein Mediates the Association Between Rheumatoid Arthritis and Atlantoaxial Subluxation,” Frontiers in Endocrinology 13 (2022): 1054206, 10.3389/fendo.2022.1054206. 36589832 PMC9800511

[22] P. Gatarek and J. Kaluzna‐Czaplinska , “Trimethylamine N‐Oxide (TMAO) in Human Health,” EXCLI Journal 20 (2021): 301–319, 10.17179/excli2020-3239. 33746664 PMC7975634

[23] Y. Li , A. Gray , L. Xue , et al., “Metabolomic Profiles, Ideal Cardiovascular Health, and Risk of Heart Failure and Atrial Fibrillation: Insights From the Framingham Heart Study,” Journal of the American Heart Association 12, no. 12 (June 2023): e028022, 10.1161/jaha.122.028022. 37301766 PMC10356055

[24] Q. Li , X. Sun , K. Yu , et al., “Enterobacter Ludwigii Protects DSS‐Induced Colitis Through Choline‐Mediated Immune Tolerance,” Cell Reports 40, no. 9 (August 2022): 111308, 10.1016/j.celrep.2022.111308. 36044853

[25] M. Ezeani and S. Elom , “Necessity to Evaluate PI3K/Akt Signalling Pathway in Proarrhythmia,” Open Heart 4, no. 2 (2017): e000596, 10.1136/openhrt-2017-000596. 29259786 PMC5729307

[26] A. Ajoolabady , S. Nattel , G. Y. H. Lip , and J. Ren , “Inflammasome Signaling in Atrial Fibrillation,” Journal of the American College of Cardiology 79, no. 23 (June 2022): 2349–2366, 10.1016/j.jacc.2022.03.379. 35680186 PMC8972346

[27] F. Müller , A. Sharma , J. König , and M. F. Fromm , “Biomarkers for In Vivo Assessment of Transporter Function,” Pharmacological Reviews 70, no. 2 (April 2018): 246–277, 10.1124/pr.116.013326. 29487084

[28] D. F. Covey , A. S. Evers , Y. Izumi , J. L. Maguire , S. J. Mennerick , and C. F. Zorumski , “Neurosteroid Enantiomers as Potentially Novel Neurotherapeutics,” Neuroscience & Biobehavioral Reviews 149 (June 2023): 105191, 10.1016/j.neubiorev.2023.105191. 37085023 PMC10750765

[29] B. J. Lee and N. A. Afshari , “Advances in Drug Therapy and Delivery for Cataract Treatment,” Current Opinion in Ophthalmology 34, no. 1 (June 2023): 3–8, 10.1097/icu.0000000000000910. 36484206

[30] S. K. Das , H. C. Ainsworth , L. Dimitrov , et al., “Metabolomic Architecture of Obesity Implicates Metabolonic Lactone Sulfate in Cardiometabolic Disease,” Molecular Metabolism 54 (December 2021): 101342, 10.1016/j.molmet.2021.101342. 34563731 PMC8640864

[31] P. Büttner , M. Bahls , R. H. Böger , et al., “Arginine Derivatives in Atrial Fibrillation Progression Phenotypes,” Journal of Molecular Medicine 98, no. 7 (July 2020): 999–1008, 10.1007/s00109-020-01932-9. 32504111 PMC8556202

[32] K. Nitz , M. Lacy , M. Bianchini , et al., “The Amino Acid Homoarginine Inhibits Atherogenesis by Modulating T‐Cell Function,” Circulation Research 131, no. 8 (September 2022): 701–712, 10.1161/circresaha.122.321094. 36102188

